# Intrahepatic cholangiocarcinoma in a patient with Wilson’s disease: a case report

**DOI:** 10.1186/s40792-016-0156-3

**Published:** 2016-03-23

**Authors:** Yosuke Mukai, Hiroshi Wada, Hidetoshi Eguchi, Daisaku Yamada, Tadafumi Asaoka, Takehiro Noda, Koichi Kawamoto, Kunihito Gotoh, Yutaka Takeda, Masahiro Tanemura, Koji Umeshita, Yumiko Hori, Eiichi Morii, Yuichiro Doki, Masaki Mori

**Affiliations:** Department of Gastroenterological Surgery, Graduate School of Medicine, Osaka University, 2-2-E2, Yamadaoka, Suita, Osaka, 565-0871 Japan; Department of Surgery, Kansai Rosai Hospital, Osaka, Japan; Department of Surgery, Osaka Police Hospital, Osaka, Japan; Division of Health Sciences, Graduate School of Medicine, Osaka University, Osaka, Japan; Department of Diagnostic Pathology, Graduate School of Medicine, Osaka University, Osaka, Japan

**Keywords:** Wilson’s disease, Intrahepatic cholangiocarcinoma, Liver tumor, Hepatobiliary malignancies

## Abstract

The incidence of hepatobiliary malignancies, and especially intrahepatic cholangiocarcinoma (ICC), for patients with Wilson’s disease (WD), is very low, even for cirrhotic patients. A 44-year-old male was admitted to our department for treatment of a liver tumor. He was diagnosed with WD at the age of 15. According to radiological findings, his liver tumor was a suspected hepatocellular carcinoma (HCC) or a combined hepatocellular and cholangiocellular carcinoma. A partial resection of liver segments 8 (S8) and 5 (S5) was subsequently performed due to the intraoperative suspicion of intrahepatic metastasis at the surface of S5. Postoperative histology revealed that the resected portion of S8 contained an ICC; the removed S5 portion comprised a regenerative nodule with hemosiderosis. To date, the patient has survived without tumor recurrence for more than 44 months following surgery. A survey of the literature, inclusive of case reports, would suggest that surgical resection is the primary course of action for a WD patient with ICC, if liver function can be preserved and curative resection performed.

## Background

Wilson’s disease (WD) is an autosomal recessive genetic disorder that results in excessive copper accumulation in the liver, brain, and other vital organs. This disorder manifests clinically as liver damage, with neurological or psychiatric symptoms. While copper overload plays a critical role in the initial liver injury that eventually leads to chronic inflammation and cirrhosis, its direct oncogenic potential remains unknown and merits further investigation [1]. Curiously, the incidence of hepatobiliary malignancies for individuals with Wilson’s disease is very low, even in cirrhotic patients [2]; occasional cases of hepatocellular carcinoma (HCC) have been reported, with the incidence of intrahepatic cholangiocarcinoma (ICC) rarer still. We now report such a case, which was successfully resected, and discuss our conclusions with reference to the current literature.

## Case presentation

**Fig. 1:**
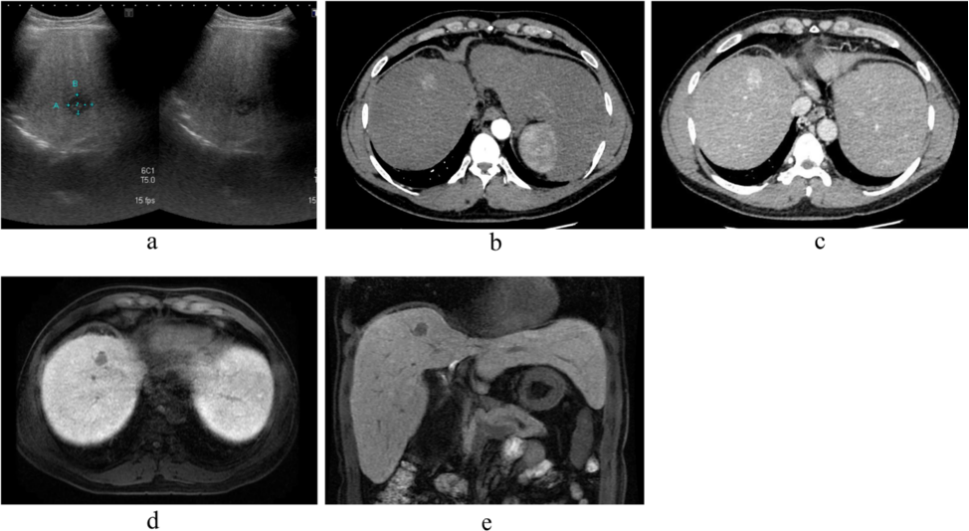
Preoperative radiological findings. **a** Abdominal ultrasonography showed a hypoechoic lesion in segment 8 (S8) of the liver. **b** The main tumor (S8) showed an early enhancement in CT, which was prolonged until the delayed phase (**c**). **d** Gadolinium ethoxybenzyl diethylenetriaminepentaacetic acid (Gd-EOB-EDTA)-enhanced MRI revealed a solitary 1.8-cm tumor, with a low-intensity signal in the hepatobiliary phase in the transverse (**d**) and sagittal planes (**e**)

**Fig. 2:**
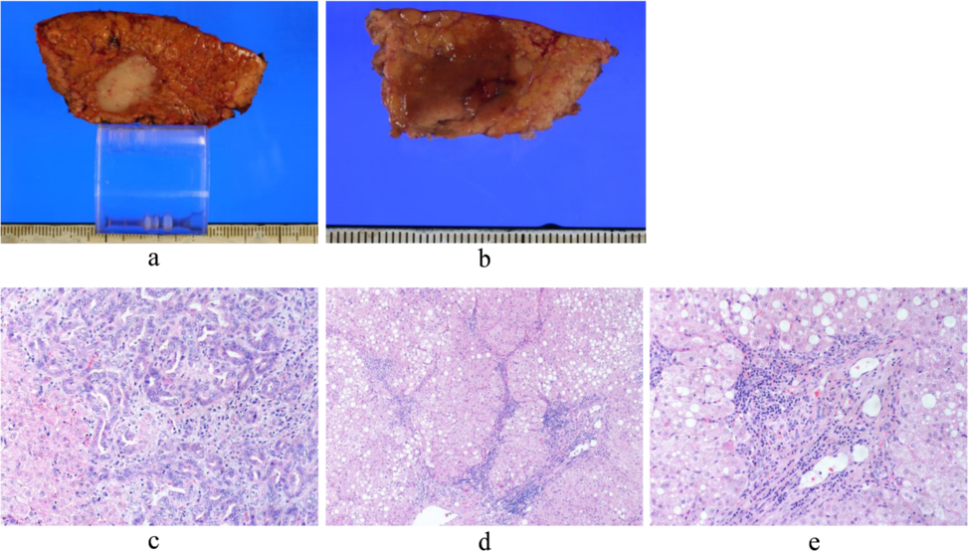
The resected specimens of S8 (**a**) and S5 (**b**). Histological examination of the resected specimens. **c** The lesion in S8 was a moderately differentiated cholangiocarcinoma of the liver (HE staining, ×100). The adjacent, non-cancerous liver parenchyma demonstrated typical cirrhotic features with steatosis (HE staining, ×40) (**d**), (HE staining, ×100) (**e**)

A 44-year-old male was admitted to our department for treatment of a liver tumor. Abnormal liver function and a subsequent blood test led to his diagnosis of WD at the age of 15; his serum levels of ceruloplasmin were barely detectable (≤20 mg/dL), with the urinary excretion of copper at 209 μg/day (≥100 μg/day). A family history showed that his sister had also been diagnosed and treated for WD. Our patient had been treated with d-penicillamine for 20 years. In the 20th year after his diagnosis, magnetic resonance imaging (MRI) revealed a solitary liver tumor, 1.8 cm in diameter, located in segment 8 (S8) of the liver. The results of laboratory examination, including liver function, were as follows: serum aspartate aminotransferase (AST) level, 87 IU/L; serum alanine aminotransferase (ALT) level, 115 IU/L; alkaline phosphatase (ALP) level, 289 IU/L; γ-glutamyl transpeptidase (γ-GTP) level, 909 IU/L; total bilirubin level, 0.5 mg/dL; prothrombin time (PT) 78 s; albumin 3.9 g/dL; and indocyanine green retention 15 min (ICG-R15), 8 %. Tests for hepatitis B surface antigen (HBs-Ag) and anti-hepatitis C virus antibody (HCV-Ab) were negative. The serum level of alpha-fetoprotein (AFP) was 37 ng/mL, and the serum levels of proteins induced by the absence of vitamin K (PIVKA-II), CA19-9, and CEA were in the normal range. Abdominal ultrasonography showed a hypoechoic lesion in S8 of the liver (Fig. 1a). By computed tomography (CT), the tumor showed an early enhancement (Fig. 1b), without a late washout pattern (Fig. 1c). In gadolinium ethoxybenzyl diethylenetriaminepentaacetic acid (Gd-EOB-EDTA)-enhanced MRI, the tumor showed a low signal intensity in the hepatobiliary phase (Fig. 1d, e). According to the radiological findings, our preoperative diagnosis was a suspected HCC or a combined hepatocellular and cholangiocellular carcinoma. During a 3-h and 46-min operation, a non-anatomical partial resection of S8 and S5 was completed because of the suspicion of intrahepatic metastasis at the surface of segment 5 (S5), which was detected as a 7-mm-sized hypoechoic lesion in the intraoperative ultrasonography. The gross appearance of liver was macro nodular cirrhosis, and its surface was irregular and its consistency was firm. Intraoperative blood loss was 200 mL. Postoperative histological examination revealed that the resected S8 lesion was an ICC (Fig. 2a), with the S5 resection containing a regenerative nodule with hemosiderosis (Fig. 2b). The resected ICC was macroscopically a whitish mass, with a clear boundary. Microscopically, the tumor cells had formed glandular structures, which led to a diagnosis of a moderately differentiated cholangiocellular carcinoma of the liver (Fig. 2c). While confirming a clean margin, the presence of pseudo-lobules with fatty change in the adjacent non-cancerous portion confirmed a diagnosis of liver cirrhosis (Fig. 2d, e). We performed the rhodamine staining to evaluate the accumulation of cooper in the non-cancerous liver, but copper accumulation was little in the resected liver specimen. The patient was discharged from the hospital without postoperative complications. At present, he is alive, without disease recurrence, for more than 44 months after his operation.

### Discussion

Wilson’s disease is a hereditary disorder of copper metabolism that results in the accumulation of copper in the body, primarily in the liver, brain, and other vital organs. Copper accumulation causes chronic liver damage and leads to cirrhosis. Although chronic liver damage is one of most important risk factors for the development of primary liver cancer, its incidence seems to be rare in WD. While the reasons for this low incidence remains obscure, it has been suggested that excess copper in the liver is protective against carcinogenesis, through stabilization of cellular chromatin [3–5]. This conclusion would appear to be at odds with a second report showing that excess copper provokes DNA damage via the generation of reactive oxygen species [6]. Clearly, the relationship between WD and liver cancer remains controversial [7, 8].

A multicenter cohort study of 1186 patients with WD revealed that their incidence of primary liver cancer was 0.28 per 1000 person years, with a prevalence of 1.2 %. The incidences of HCC and ICC for individuals with WD are 0.7 and 0.5 %, respectively [2]. In contrast, the rate of HCC development in patients who have contracted the hepatitis C virus is 3.9 % per year; the risk of primary liver cancer for individuals with WD is therefore demonstrably low.

Intrahepatic cholangiocarcinoma (ICC) is the second most common primary liver cancer [9], and has been considered incurable, and rapidly lethal unless full lesional resection can be achieved. The prognosis of biliary tract cancer also remains poor [10]. Twelve cases of ICC associated with WD have been reported in the literature, including the present case study (Table 1); six of these cases were from the Pfeiffenberger cohort study [2], with a further three cases from Walshe’s cohort study [11], although details for these cases were not included. Of the two remaining reports [12, 13], one described a patient undergoing liver transplantation for WD, with a small nodule, which was treated with four cycles of doxorubicin-based, transcatheter arterial chemoembolization. The tumor was subsequently diagnosed as an ICC in the explanted liver. Due to recurrent ICC after liver transplantation, selective internal radiation therapy was performed followed by right hemihepatectomy [12]. The final report described a diagnosis of WD following a right hepatic lobectomy and lymph node dissection, for a patient with WD [13]. In the present case, the tumor was about 4 cm in diameter in S8 of the liver. Histologic examination showed that the tumor was a moderately differentiated cholangiocarcinoma and that the non-cancerous liver was fibrotic, with steatosis.

**Table 1:** Clinical characteristics of patients with Wilson’s disease

References	Sex	Age at tumor diagnosis	Time until tumor detection from diagnosis of WD (years)	Therapy for ICC	Patient status
Walshe (2003) [11]	F	28	15	–	Not available
Walshe (2003) [11]	F	40	24	–	Not available
Walshe (2003) [11]	F	85	22	–	Not available
Saito (2009) [13]	M	39	28	Right lobectomy	Not available
Sperling (2014) [12]	–	55	–	Liver transplantation for primary tumor (radiation and right hemihepatectomy for recurrence)	48 months, alive
Pfeiffenberger (2015) [2]	M	53	22	Orthotopic liver transplantation, chemotherapy (GEM/CDDP)	26 months, dead
Pfeiffenberger (2015) [2]	M	43	19	Chemotherapy (GEM)	12 months, dead
Pfeiffenberger (2015) [2]	F	33	6	Chemotherapy (CDDP/GEM)	lost for follow-up
Pfeiffenberger (2015) [2]	M	72	41	Surgical resection	Not available
Pfeiffenberger (2015) [2]	M	56	7	RFA, chemotherapy (CDDP/GEM)	12 months, dead
Pfeiffenberger (2015) [2]	F	70	41	Surgical resection	Not available
Present case	M	44	20	Surgical resection	44 months, alive

*GEM* gemcitabine, *CDDP* cisplatin

Five of the 12 cases, including ours, were treated with surgical resection; all of these patients were alive at the time of writing. Recently, the Mayo clinic reported that liver transplantation following high-dose neoadjuvant radiotherapy with chemosensitization achieved excellent results for patients with early stage, unresectable hilar cholangiocarcinoma or cholangiocarcinoma arising in the setting of primary sclerosing cholangitis [14]. Liver transplantation is reported as one of treatment options for WD, for which there is an excellent long-term outcome. However, liver failure associated with WD remains a rare indication for liver transplantation (less than 1 %).

## Conclusions

In the present case, we performed a partial liver resection for ICC; the patient has survived without tumor recurrence in excess of 44 months following curative resection. Surgical resection would therefore seem to be the treatment of choice for a WD patient with ICC, when liver function can be preserved and curative resection performed.

## Consent

Written informed consent was obtained for this publication and accompanying images, from the patient involved. A copy of this written consent is available for review by the Editor-in-Chief of this journal.

## Abbreviations

AFP: alpha-fetoprotein
ALP: alkaline phosphatase
ALT: alanine aminotransferase
AST: aspartate aminotransferase
CT: computed tomography
Gd-EOB-EDTA: gadolinium ethoxybenzyl diethylenetriaminepentaacetic acid
HBs-Ag: hepatitis B virus surface antigen
HCC: hepatocellular carcinoma
HCV-Ab: hepatitis C virus antibody
ICC: intrahepatic cholangiocarcinoma
ICG-R15: indocyanine green retention 15 min
MRI: magnetic resonance imaging
PIVKA-II: protein induced by the absence of vitamin K
S: segment
WD: Wilson’s disease
γ-GTP: γ-glutamyl transpeptidase
